# The Teeth and the Skull

**Published:** 1912-08-15

**Authors:** 


### THE TEETH AND THE SKULL.

It occasionally happens that physiologic forces which are active in the organism are not adequately recognized until some pathologic condition makes their normal presence suspected. The teeth are ordinarily looked on merely as organs for mastication exerting a purely local influence in the mouth and unsuspected as agents whose proper development is of considerable significance for the growth of the entire skull. In the course of the development of the teeth within the jaws from their delicate beginnings as tooth-sacs until their final evolution into perfect dental organs they must inevitably be demanding more and more room and accordingly be exerting a certain degree of tension or pressure on the parts within which they are growing. Does this expansion exert any directive influence on the development of the jaws as a whole, or is the evolution of the teeth merely a localized incident in the changing form of the jaws with the progress of age? This question is one which is amenable to experimental treatment. Dr. Landsberger of Berlin has watched the effects produced by the extirpation of the tooth-sacs of both the permanent and the milk teeth before they have developed sufficiently to exert any influence on their bony environment.* In this way he was able to distinguish between the inherent growth impulse

*Landsberger, R.: Der Einfluss der Zaehne auf die Entwickelung des Schaedels, *Arch. f. Physiol.*, 1911, p. 433.

in the jaws themselves and the possible contribution of the teeth to the contour and enlargement of the bony parts.

Such extirpations of the tooth-sacs, made on young dogs, were always undertaken on one-half of the jaw, so that comparisons could be made with the side unoperated on. An entire year was allowed to elapse before the skulls were prepared for examination. The changes observed were typical and constant. The direction of growth in the jaw was always altered so that a twisted or one-sided effect difficult to describe adequately in words was obtained; there were also evidences of inadequate development in certain parts of the bony skull; and a marked hypertrophy of certain parts about the nostrils was prominently in evidence.

The "twist" in the upper jaw occasioned by the absence of growing teeth calls for some comment. With the lack of tension or pressure ordinarily exerted by the developing tooth-sacs on one side, nothing interferes to oppose the strain on the other side. A distortion of skeletal form ensues as a consequence. This does not make itself manifest on the lower jaw, because it is equalized by the movability of the attachments of this part.

The interference with the well-proportioned development of the skull as a whole—a distortion in the vertical direction of the half operated on and a spreading in a horizontal way of the side not operated on—points to influences on growth exerted far beyond the immediate locality of the teeth. This unanticipated interruption of perfect skull development as the result of imperfect organization of the teeth may have wider import for medical problems than appears at first glance. If, as Landsberger remarks, improper development of the teeth can influence the skull in a pathologic way, as these animal experiments clearly indicate, it is not beyond the bounds of reason to suppose that the brain functions may also become involved more or less. Nervous upsets associated with the difficulties of dentition are not unknown in practice; and the apparent correlations between teeth and skull may have a bearing for both the psychiatrist and the rhinologist. Traction devices applied by dental surgeons in cases of defects in dental development are reputed to have improved both the mental and physical condition of children.

Perhaps we have, in the past, failed to recognize the importance of the growing teeth in lending appropriate stimulus to the correct anatomic development of the head. Stomatology must become an experimental science—something more than an odd-sounding name of vague import.—*Ed. Journal A. M. A.*, June 15, 1912.

